# The Potential of Fecal and Urinary Biomarkers for Early Detection of Pancreatic Ductal Adenocarcinoma: A Systematic Review

**DOI:** 10.7759/cureus.59248

**Published:** 2024-04-29

**Authors:** Talha Arif, Faran Nazir, Raja F Aurangzeb, Mubashir Hussain, Raja I Aurangzeb, Abdur Rehman, Kabeer Kumar, Rabia Islam, Hamza Islam, Qais Khalid, Divine B Arrey Agbor, Kashaf Munir, Syed Faqeer H Bokhari, Abdullah Shehryar, Muhammad Ibrahim

**Affiliations:** 1 Accident and Emergency, Imran Idrees Teaching Hospital, Sialkot, PAK; 2 Internal Medicine, Faisalabad Medical University, Deer Park, USA; 3 Internal Medicine, Fauji Foundation Hospital, Rawalpindi, PAK; 4 Internal Medicine, Chandka Medical College, Larkana, PAK; 5 Surgery, Mayo Hospital, Lahore, PAK; 6 Research, Faisalabad Medical University, Faisalabad, PAK; 7 Internal Medicine, Punjab Medical College, Faisalabad, PAK; 8 Internal Medicine, Khyber Medical University, Peshawar, PAK; 9 Internal Medicine, Richmond University Medical Center, Staten Island, USA; 10 Medicine, Shalamar Medical and Dental College, Lahore, PAK; 11 Surgery, King Edward Medical University, Lahore, PAK; 12 Internal Medicine, Allama Iqbal Medical College, Lahore, PAK; 13 Medicine, Jinnah Hospital, Lahore, PAK

**Keywords:** fecal biomarkers, urinary biomarkers, git, pancrease, pdac (pancreatic ductal adenocarcinoma)

## Abstract

Pancreatic ductal adenocarcinoma (PDAC) is a highly lethal cancer often diagnosed at advanced stages, highlighting the urgent need for early detection strategies. This systematic review explores the potential of fecal and urinary biomarkers for early PDAC detection. A comprehensive search identified eight relevant studies investigating various biomarkers, including proteins, metabolites, microbial profiles, DNA mutations, and non-coding RNAs. Promising findings suggest that urinary biomarkers related to metabolic alterations, inflammatory processes, fecal microbiome profiles, and fecal miRNAs hold diagnostic potential even at early stages of PDAC. Combining biomarkers into panels may enhance diagnostic accuracy. Challenges such as validation in larger cohorts, standardization of protocols, and regulatory approval must be addressed for clinical translation. Despite these hurdles, non-invasive urinary and fecal biomarkers represent a promising avenue for improving PDAC outcomes through early detection.

## Introduction and background

Pancreatic ductal adenocarcinoma (PDAC) is one of the most lethal cancers, with a five-year survival rate of only 9% [1]. Most patients are diagnosed at an advanced stage when curative surgery is no longer possible, contributing to the dismal prognosis [2]. Early detection of PDAC is critical to improve patient outcomes, as surgical resection at an early stage offers the only potential cure [3]. In recent years, research has focused on identifying biomarkers in biofluids other than blood, such as urine and stool, which may detect molecular changes associated with early carcinogenesis. These non-invasive tests have the advantages of being cost-effective, reproducible, and convenient for patients [4]. Several potential fecal and urinary biomarkers for early PDAC detection have been identified, including proteins, metabolites, DNA mutations, and microRNAs (miRNAs) [5].

Urinary biomarkers are attractive candidates as urine collection is simple, and urinary molecular changes may reflect systemic alterations during PDAC development [6]. Urinary metabolites related to glucose metabolism, inflammation, and cachexia have shown potential for early PDAC detection [7]. Urinary exosomal miRNAs, stable, extracellular miRNAs encapsulated in vesicle nanoparticles, have also shown diagnostic potential [8].

Likewise, fecal biomarkers are promising as molecular alterations in pancreatic secretions may be detectable in stool long before symptoms arise [9]. Fecal microbial profiles are altered in PDAC patients, and PDAC can be distinguished from healthy controls [10]. Specific bacterial species and fecal volatile organic compounds may serve as early diagnostic indicators of PDAC [11]. Additionally, differential expression of fecal miRNAs has been detected in PDAC patients compared to healthy individuals [12].

In summary, evidence suggests that fecal and urinary biomarkers could enable earlier PDAC detection than currently possible. Non-invasive urine and stool tests present a novel screening and early diagnosis approach. Further, prospective studies are warranted to validate the clinical utility of these biomarkers. Systematic reviews summarizing the current evidence on these emerging biomarkers are important to guide future research and facilitate potential translation into clinical practice for earlier PDAC detection and improved patient outcomes.

## Review

Material and methods

Search Strategy

Our search strategy was developed in line with the Preferred Reporting Items for Systematic Reviews and Meta-Analysis (PRISMA) guidelines to ensure the thorough identification of studies pertinent to fecal and urinary biomarkers for early detection of pancreatic ductal adenocarcinoma (PDAC). Extensive searches were performed across several electronic databases, including PubMed, Medline, Embase, and the Cochrane Library, with the timeframe extending from the inception of each database to December 2023.

The search strategy integrated keywords and Medical Subject Headings (MeSH) terms relevant to our research question. Terms such as 'pancreatic ductal adenocarcinoma', 'fecal biomarkers', 'urinary biomarkers', 'early detection', and 'diagnostic performance' were included. Boolean operators were employed to enhance the search, for example, 'pancreatic ductal adenocarcinoma AND fecal biomarkers' or 'urinary biomarkers AND early detection.'

Additionally, we tailored the search to capture studies on various biomarkers studied in the context of PDAC, such as 'creatinine', 'LYVE1', 'REG1B', 'TFF1', 'microbiome signatures', 'MIR1246', 'trigonelline', 'glycolate', 'hippurate', 'creatine', 'myoinositol', 'hydroxyacetone', 'miR-21', 'miR-155', 'miR-196a', 'miR-216', and 'miR-217'. These terms were combined with diagnostic performance metrics like 'accuracy', 'sensitivity', and 'specificity'.

To ensure the inclusion of grey literature and ongoing research, our strategy extended to scrutinizing the reference lists of selected articles and exploring clinical trial registries and relevant conference proceedings. The comprehensiveness of our search was validated by a medical information retrieval specialist, confirming the alignment of our methodology with the PRISMA guidelines.

Eligibility Criteria

The eligibility criteria for our systematic review were meticulously defined to ensure the selection of relevant and high-quality studies. Our inclusion criteria are tailored to peer-reviewed research articles, case-control studies, cohort studies, and clinical trials that examine fecal and urinary biomarkers in early pancreatic ductal adenocarcinoma (PDAC) detection. We consider studies that evaluate the diagnostic performance of these biomarkers, including metrics such as accuracy, sensitivity, and specificity, and those that focus on developing diagnostic algorithms or models.

Our review encompasses studies focusing on three main pancreatic ductal adenocarcinoma (PDAC) objectives. Firstly, we examine research exploring the correlation between fecal or urinary biomarkers and early-stage PDAC to elucidate potential diagnostic indicators. Secondly, we evaluate the feasibility of implementing these biomarkers in clinical environments for the purpose of screening individuals for PDAC, thereby potentially enhancing early detection rates. Lastly, we prioritize studies that rigorously validate these biomarkers' efficacy by ensuring adequate control groups' presence, thereby enhancing the reliability and robustness of the findings. Through this comprehensive analysis, we aim to contribute insights that could inform advancements in PDAC detection and management strategies. Only studies published in English were included to ensure consistency in the review process.

Conversely, our exclusion criteria remove articles that do not directly assess fecal or urinary biomarkers for PDAC. Studies focusing solely on other forms of cancer or non-cancerous pancreatic diseases were excluded to maintain a clear focus on PDAC. Similarly, articles based on animal models, case reports, editorials, commentary, and grey literature, including conference abstracts, posters, and unpublished works, were also excluded. This decision ensured that the data synthesized in our review came from studies with the most robust methodological designs relevant to human clinical outcomes in the context of early PDAC detection.

By adhering to these eligibility criteria, our review ensures the inclusion of studies that are most pertinent to advancing the understanding and clinical application of fecal and urinary biomarkers in the early detection of PDAC.

Data Extraction

A systematic data extraction methodology has been implemented to guarantee accuracy and thoroughness in our systematic review. The initial phase involves an assessment of articles based on titles and abstracts. This task is executed by two independent reviewers who categorize each article as "relevant", "probably relevant", or "not relevant". Such a preliminary filtering stage is fundamental to identifying articles that warrant more detailed examination.

Following this, full-text articles classified as "relevant" or "probably relevant" are retrieved for comprehensive evaluation. Data from these articles is extracted using a standardized data collection template created in Microsoft Excel (Microsoft, Redmond, Washington). This approach promotes the consistency of the data extraction across all studies.

In parallel, the independent reviewers utilize the predefined inclusion and exclusion criteria to assess each article's eligibility. When discrepancies between the two reviewers arise, they are resolved by consulting a third independent reviewer, ensuring a consensus is reached and thus maintaining the integrity of the data extraction process.

The data extraction template is meticulously designed to capture essential information pertinent to our research question. It includes fields for author(s), year of publication, biomarker(s) studied, study design, population size, and key metrics of diagnostic performance such as accuracy, sensitivity, and specificity. Moreover, it collates the primary findings and any recommendations made by the study. This detailed data extraction procedure enables the construction of a comprehensive database, facilitating subsequent analysis and synthesis of the extracted data and ensuring no significant detail is overlooked.

This rigorous data extraction method ensures that the conclusions drawn from our systematic review are based on a complete and accurate representation of the available evidence concerning fecal and urinary biomarkers for early detection of pancreatic ductal adenocarcinoma (PDAC).

Data Analysis and Synthesis

Our review's data analysis and synthesis are structured to methodically evaluate the diagnostic potential of fecal and urinary biomarkers in early pancreatic ductal adenocarcinoma (PDAC) detection. Quantitative data, such as diagnostic performance measures, sensitivity, specificity, and accuracy rates, will be statistically analyzed. Where data homogeneity permits, a meta-analysis will be considered to pool performance metrics and provide a more robust estimation of biomarker efficacy.

Qualitative data encompassing study findings and authors' recommendations will undergo thematic synthesis. This process involves extracting themes related to these biomarkers' utility, applicability, and potential integration into clinical practice.

Integrating quantitative and qualitative analyses will offer a comprehensive view of the current state of fecal and urinary biomarkers in PDAC diagnosis. A narrative synthesis will complement the statistical findings, contextualizing them within the broader research landscape and discussing their implications in line with the existing body of knowledge.

In addition, we will evaluate the evidence's quality and the strength of the synthesized data, identifying any literature gaps or inconsistencies. By adopting this analytical approach, our synthesis aims to provide a concise yet comprehensive understanding of the early detection capabilities of fecal and urinary biomarkers for PDAC, ultimately contributing to advancing non-invasive diagnostic methods in oncology.

Results

Study Selection Process

Our systematic search across multiple electronic databases initially retrieved 121 articles. Upon removing 27 duplicates, we screened 94 records by title and abstract. The screening process was guided by our inclusion criteria, which required studies to focus on fecal and urinary biomarkers to detect PDAC early and to report on diagnostic performance outcomes such as accuracy, sensitivity, and specificity. We excluded studies that were not in English, did not pertain to human clinical outcomes, or focused on other types of cancer or diseases. Studies were also excluded if they were based on animal models, were reviews or editorials, or were grey literature, such as conference abstracts or unpublished studies.

After the screening process, 78 records were excluded, leaving 16 reports that were sought for full-text retrieval. Of these, 13 reports could be retrieved and were subjected to a rigorous assessment for eligibility based on the above criteria, excluding five more studies.

**Figure 1:**
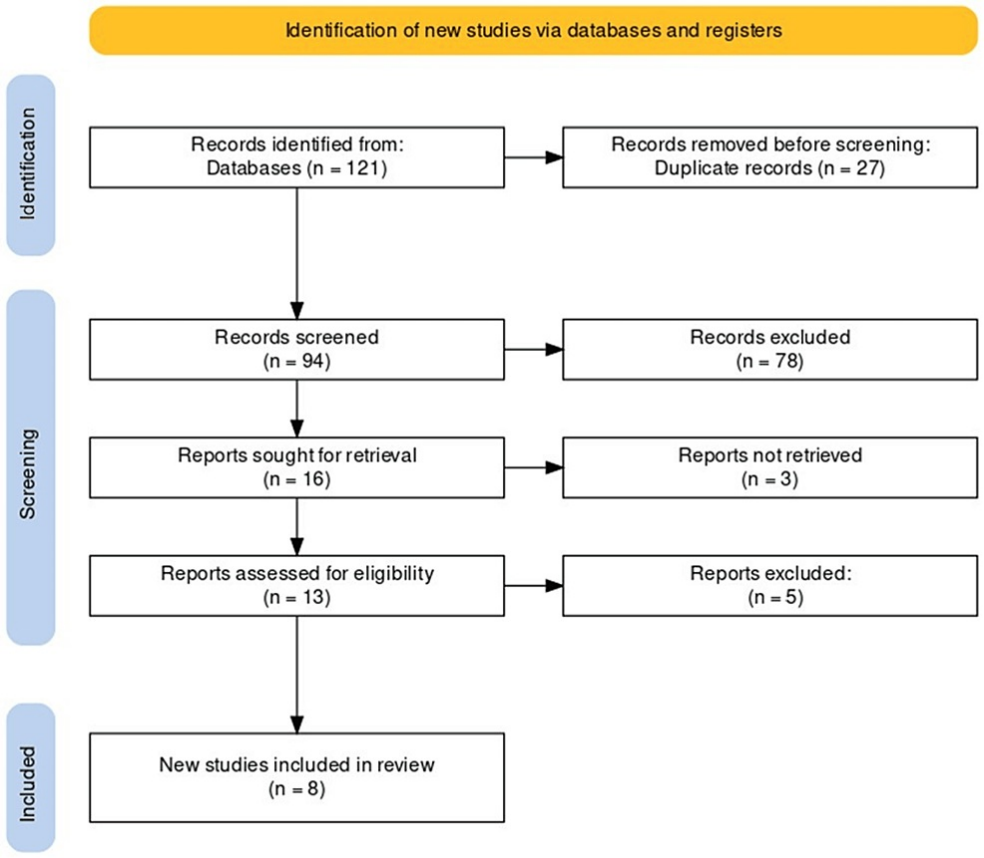
PRISMA flow diagram of selection of studies for inclusion in the systematic review PRISMA - Preferred Reporting Items for Systematic Reviews and Meta-Analysis (PRISMA)

Consequently, eight new studies met our inclusion criteria and were selected for this review. The reference lists of these studies were scrutinized for potentially relevant studies, but this did not yield any additional articles for inclusion. The entire process, from identification to the final inclusion of studies, is depicted in the PRISMA flowchart (Figure 1). This visual aids in transparently illustrating the systematic approach and meticulous criteria we adhered to in selecting studies for our review.

Characteristics of Selected Studies

Our systematic review incorporates eight significant studies that provide valuable information regarding fecal and urinary biomarkers for the early detection of PDAC. The selected studies encompass various research methodologies, including developing diagnostic models, validating biomarker panels, and analyzing biomarker efficacy using different patient cohorts and control groups.

These studies were published between 2014 and 2023 and utilized various analytical techniques, from deep learning models to metagenomic sequencing and mass spectrometry. The patient populations vary in size, from smaller, focused cohorts to larger, more comprehensive collections of samples. The diagnostic performance of the biomarkers studied is quantitatively presented with accuracy, sensitivity, specificity, and other relevant metrics.

A summary of these studies, including the authors, publication year, biomarkers studied, study design, population size, and main findings, is concisely presented in Table 1. This table is an accessible guide to grasp the diversity and depth of research conducted in this field, providing a solid foundation for a nuanced understanding of the potential of fecal and urinary biomarkers in the early diagnosis of PDAC.

**Table 1:** A summary of the studies included in this systematic review. PDAC - pancreatic ductal adenocarcinoma; NAFLD - nonalcoholic fatty liver disease; PC - pancreatic cancer; PCL - pre-cancerous lesion; ELISA - enzyme-linked immunosorbent assay

Author	Year	Biomarker(s) studied	Study design	Population size	Diagnostic performance (e.g., accuracy, sensitivity, specificity)	Main findings and recommendations
Karar et al. [13]	2023	Creatinine, LYVE1, REG1B, TFF1	Development of 1D CNN-LSTM model	Not specified	Accuracy: 97%	Developed a deep learning model with high accuracy for classifying PDAC. Suggests the promising utility of urinary biomarkers.
Kartal et al. [10]	2022	Fecal microbiome signatures	Shotgun metagenomic and 16S rRNA sequencing	Spanish case-control: 136, German validation: 76	Accuracy up to 0.84, improved to 0.94 with CA19-9 levels	Identified fecal microbiota signatures with high disease specificity. Supports fecal microbiota-based screening.
Debernardi et al. [14]	2020	LYVE1, REG1B, and TFF1	Panel validation and development of the PancRISK algorithm	Larger cohort of retrospectively collected samples	Specificity and sensitivity: >85%	Demonstrated the potential of a urinary biomarker panel for early PDAC detection and risk stratification.
Ishige et al. [15]	2020	MIR1246	Analysis of serum and urine levels	Not specified	Sensitivity: 92.3% (serum), 90.2% (urine); Specificity: 73.3% (serum), 83.3% (urine)	Suggested MIR1246 as a promising biomarker. Recommends further validation in larger cohorts.
Sahni et al. [16]	2020	Trigonelline, Glycolate, Hippurate, Creatine, Myoinositol, Hydroxyacetone	Metabolite panel evaluation	Discovery and validation cohorts	AUROC: 0.933 (discovery), 0.864 (validation)	Showed potential of metabolites for early-stage PDAC detection. Highlights the need for non-invasive biomarkers.
Half et al. [17]	2019	Fecal microbiome signatures	Deep amplicon sequencing of 16S rRNA gene	PC: 30, NAFLD: 16, PCL: 6, vontrols: 13	Not specified	Identified microbial patterns associated with PC. Emphasizes the importance of a multi-marker approach.
Radon et al. [6]	2015	LYVE-1, REG1A, TFF1	GeLC/MS/MS analysis and ELISA validation	Not specified	AUCs: 0.891 (training), 0.921 (validation)	Found biomarker panel effective for early-stage PDAC detection. Advocates for combining biomarkers for improved accuracy.
Yang et al. [12]	2014	miR-21, miR-155, miR-196a, miR-216, miR-217	Analysis of miRNAs in clinical specimens	Not specified	High sensitivity and specificity	Identified stool miRNAs as potential non-invasive biomarkers for PDAC screening. Recommends combining miR-21, miR-155, and miR-216 for diagnostics.

Discussion

This systematic review has consolidated the evidence on the burgeoning field of fecal and urinary biomarkers for the early detection of pancreatic ductal adenocarcinoma (PDAC), a malignancy notorious for its late presentation and poor prognosis. The discovery of multiple biomarker candidates spanning various biological substrates, such as metabolites, proteins, microbial profiles, DNA mutations, and non-coding RNAs, heralds a new era in the non-invasive detection of this lethal cancer. Historically, the diagnostic journey for PDAC has been fraught with obstacles, mainly due to the absence of specific symptoms and the rapid progression of the disease. Conventional diagnostic tools, such as imaging and tissue biopsies, often only confirm the disease at an advanced stage when therapeutic options are limited and less effective. In contrast, the biomarkers identified in our review could signify a paradigm shift, promising a timely and more precise diagnosis. By potentially detecting the disease when it is still localized within the pancreas, these biomarkers could dramatically improve survival rates by enabling curative surgical interventions and personalized therapeutic strategies.

Urinary biomarkers related to metabolic alterations, inflammation, and cachexia in PDAC demonstrated discriminatory power even at early stages [6,17,18]. Certain urinary proteins involved in processes like angiogenesis and extracellular matrix remodeling were specifically upregulated in PDAC patients versus controls [5]. Urinary exosomal miRNAs also exhibited diagnostic potential, likely reflecting underlying tumor molecular characteristics [8]. Fecal microbiome profiles distinguished PDAC patients from healthy individuals [10]. Specific bacterial species and fecal volatile metabolites showed alteration even before symptom onset and could differentiate PDAC. Fecal DNA mutations shed from apoptotic cancer cells into the duodenum offered early diagnostic possibilities [19]. Differentially expressed fecal miRNA levels could also detect PDAC earlier than conventional methods [12].

The potential of these non-invasive biomarkers extends beyond the laboratory; they stand as the cornerstone for developing groundbreaking early detection strategies for PDAC, which currently has one of the lowest survival rates among cancers. The urgency of this need cannot be overstated; early-stage detection is directly correlated with increased survival, yet most patients present with advanced disease. The translation of these biomarkers from research to clinical application hinges on robust validation studies that confirm their diagnostic accuracy and evaluate their effectiveness in real-world screening, particularly in asymptomatic individuals who may benefit the most from early detection [14].

The complexity of PDAC pathobiology suggests that a multiplexed approach, leveraging a panel of biomarkers, may capture the heterogeneity of the disease better than any single marker. This synergistic approach could enhance diagnostic accuracy and specificity, thereby minimizing the risk of false positives, which is crucial in cancer screening. However, the successful implementation of such panels necessitates meticulous optimization and standardization of sample collection and processing protocols, which are paramount to ensuring the reproducibility and reliability of results across different populations and clinical settings. Furthermore, analytical platforms used to detect these biomarkers need to be validated for clinical use, a process that must be navigated alongside regulatory bodies to ensure the tests meet the stringent requirements for safety and efficacy before they can be approved for widespread use [15].

Several challenges remain before these biomarkers can be clinically implemented. Larger cohorts across multiple centers are needed to validate preliminary findings and account for potential demographic and geographic variations [20]. Standardized sample collection, storage, and analysis protocols must be established to ensure reproducibility. Appropriate cutoffs for distinguishing PDAC must be defined, which may require machine learning approaches applied to big datasets [21]. Strategies should be developed to integrate these emerging biomarkers with existing methods, such as cost-effective imaging.

Nonetheless, fecal and urinary biomarkers represent a promising new direction in PDAC early diagnosis. Development of screening approaches combining risk factors like age and smoking with biomarker testing could enable the identification of individuals for targeted investigations, potentially improving detection at resectable stages. Earlier diagnosis would also expand eligibility for emerging therapies. Ultimately, convenient, non-invasive tests using urine or stool could provide an opportunity for routine PDAC screening and detection at curable stages, leading to improved prognosis for this lethal malignancy. It is also important to determine the time during carcinogenesis when these biomarkers become detectable, as extremely early changes may reflect unviable screening targets due to low specificity [22].

Despite the considerable challenges ahead, the advent of non-invasive urinary and fecal biomarkers represents a seismic shift in the landscape of PDAC early detection. These biomarkers have the disruptive potential to revolutionize outcomes by diagnosing PDAC at stages amenable to surgical intervention, the only curative treatment option currently available. With meticulous validation against clinical endpoints and a strategy for seamless integration into existing diagnostic frameworks, these biomarkers could substantially alter the trajectory of this malignancy, shifting it from a near-certain death sentence to a condition with a hopeful prognosis.

The promise these biomarkers hold is not just in their scientific novelty but in their capacity to be integrated into routine screening programs. This could lead to earlier intervention strategies and open new avenues for personalized medicine in PDAC management. As this field matures, it is imperative that the scientific community continues to build upon the groundwork laid out by the studies in this review, overcoming obstacles from bench to clinic to harness the full potential of these biomarkers.

Therefore, the future of PDAC management may lie in the everyday, where indicators of our body's deepest health secrets are captured in what we leave behind. In what may be seen as the most unassuming places, like our toilets and trash cans, lies the key to unlocking a new chapter in the fight against one of the deadliest forms of cancer. The symbol of this future is the non-invasive nature of these diagnostic tools, embodying hope for early detection and a testament to the innovative spirit of medical research. In this regard, the continued investment and focus on the translational research of these biomarkers are not just warranted but imperative.

## Conclusions

Fecal and urinary biomarkers offer a non-invasive approach for the early detection of PDAC, addressing critical gaps in current diagnostic strategies. The synthesis of evidence from this systematic review underscores the diagnostic potential of various biomarkers, highlighting the promise of identifying PDAC at earlier, more treatable stages. However, significant challenges remain in terms of validation, standardization, and regulatory approval. Overcoming these hurdles is essential to realize the transformative potential of these biomarkers in clinical practice. As such, rigorous multi-center trials are needed to confirm their efficacy and reliability across diverse populations. Furthermore, integrating these biomarkers into existing screening programs could enhance their practical utility and acceptance in the medical community.

With continued research efforts and strategic implementation, non-invasive urinary and fecal biomarkers could revolutionize PDAC management, ultimately leading to improved patient outcomes and survival rates. Future research should also focus on the cost-effectiveness of implementing these screening tools in routine healthcare settings, which could be pivotal for their widespread adoption. Additionally, as we advance our understanding of the molecular pathways involved in PDAC, biomarkers could also aid in personalizing treatment strategies, thereby not only detecting the disease earlier but also tailoring interventions to individual patient profiles. The journey from discovery to clinical application is complex and fraught with challenges, but the potential benefits for early detection and management of PDAC could be profound, altering the course of one of the most lethal cancers.

## References

[1] Rahib L, Smith BD, Aizenberg R, Rosenzweig AB, Fleshman JM, Matrisian LM (2014). Projecting cancer incidence and deaths to 2030: the unexpected burden of thyroid, liver, and pancreas cancers in the United States. Cancer Res.

[2] Vincent A, Herman J, Schulick R, Hruban RH, Goggins M (2011). Pancreatic cancer. Lancet.

[3] Poruk KE, Gay DZ, Brown K (2013). The clinical utility of CA 19-9 in pancreatic adenocarcinoma: diagnostic and prognostic updates. Curr Mol Med.

[4] Brand RE, Nolen BM, Zeh HJ (2011). Serum biomarker panels for the detection of pancreatic cancer. Clin Cancer Res.

[5] Kaur S, Baine MJ, Jain M, Sasson AR, Batra SK (2012). Early diagnosis of pancreatic cancer: challenges and new developments. Biomark Med.

[6] Radon TP, Massat NJ, Jones R (2015). Identification of a three-biomarker panel in urine for early detection of pancreatic adenocarcinoma. Clin Cancer Res.

[7] Debernardi S, Blyuss O, Rycyk D (2023). Urine biomarkers enable pancreatic cancer detection up to 2 years before diagnosis. Int J Cancer.

[8] Madhavan B, Yue S, Galli U (2015). Combined evaluation of a panel of protein and miRNA serum-exosome biomarkers for pancreatic cancer diagnosis increases sensitivity and specificity. Int J Cancer.

[9] Miyabayashi K, Ijichi H, Fujishiro M (2022). The role of the microbiome in pancreatic cancer. Cancers (Basel).

[10] Kartal E, Schmidt TS, Molina-Montes E (2022). A faecal microbiota signature with high specificity for pancreatic cancer. Gut.

[11] Sugimoto M, Wong DT, Hirayama A, Soga T, Tomita M (2010). Capillary electrophoresis mass spectrometry-based saliva metabolomics identified oral, breast and pancreatic cancer-specific profiles. Metabolomics.

[12] Yang JY, Sun YW, Liu DJ, Zhang JF, Li J, Hua R (2014). MicroRNAs in stool samples as potential screening biomarkers for pancreatic ductal adenocarcinoma cancer. Am J Cancer Res.

[13] Karar ME, El-Fishawy N, Radad M (2023). Automated classification of urine biomarkers to diagnose pancreatic cancer using 1-D convolutional neural networks. J Biol Eng.

[14] Debernardi S, O'Brien H, Algahmdi AS (2020). A combination of urinary biomarker panel and PancRISK score for earlier detection of pancreatic cancer: a case-control study. PLoS Med.

[15] Ishige F, Hoshino I, Iwatate Y (2020). MIR1246 in body fluids as a biomarker for pancreatic cancer. Sci Rep.

[16] Sahni S, Pandya AR, Hadden WJ (2021). A unique urinary metabolomic signature for the detection of pancreatic ductal adenocarcinoma. Int J Cancer.

[17] Half E, Keren N, Reshef L (2019). Fecal microbiome signatures of pancreatic cancer patients. Sci Rep.

[18] Borrebaeck CAK, Mellby LD, King TC (2022). Biomarkers for the early detection of pancreatic ductal adenocarcinoma. Gastrointestinal Cancers.

[19] Kato S, Honda K (2020). Use of biomarkers and imaging for early detection of pancreatic cancer. Cancers (Basel).

[20] Al-Shaheri FN, Alhamdani MS, Bauer AS, Giese N, Büchler MW, Hackert T, Hoheisel JD (2021). Blood biomarkers for differential diagnosis and early detection of pancreatic cancer. Cancer Treat Rev.

[21] Wang CJ, Xu RH, Yuan QY (2013). Bioinformatics method to analyze the mechanism of pancreatic cancer disorder. J Comput Biol.

[22] Lee B, Lipton L, Cohen J (2019). Circulating tumor DNA as a potential marker of adjuvant chemotherapy benefit following surgery for localized pancreatic cancer. Ann Oncol.

